# Clonal Hematopoiesis and the Risk of Hematologic Malignancies after Curative Therapies for Sickle Cell Disease

**DOI:** 10.3390/jcm11113160

**Published:** 2022-06-02

**Authors:** Lukasz P. Gondek, Vivien A. Sheehan, Courtney D. Fitzhugh

**Affiliations:** 1 Department of Oncology, Division of Hematologic Malignancies, Sidney Kimmel Comprehensive Cancer Center, Johns Hopkins University, CRB1-290, 1650 Orleans Street, Baltimore, MD 21287, USA; lgondek1@jhmi.edu; 2 Department of Pediatrics, Emory University School of Medicine, Children’s Healthcare of Atlanta, 2015 Uppergate Drive, Atlanta, GA 30322, USA; vivien.sheehan@emory.edu; 3 Cellular and Molecular Therapeutics Branch, National Heart, Lung, and Blood Institute, NIH, 10 Center Drive, Bethesda, MD 20814, USA

**Keywords:** sickle cell disease, leukemia, allogeneic, hematopoietic cell transplant, gene therapy, clonal hematopoiesis

## Abstract

Sickle cell disease (SCD) is associated with severe morbidity and early mortality. Two large population studies found an increased risk for leukemia in individuals with SCD. Notably, while the relative risk of leukemia development is high, the absolute risk is low in individuals with SCD who do not receive cell-based therapies. However, the risk of leukemia in SCD is high after graft rejection and with gene therapy. Clonal hematopoiesis (CH) is a well-recognized premalignant condition in the general population and in patients after high-dose myelotoxic therapies. Recent studies suggest that CH may be more common in SCD than in the general population, outside the cell-based therapy setting. Here, we review risk factors for CH and progression to leukemia in SCD. We surmise why patients with SCD are at an increased risk for CH and why leukemia incidence is unexpectedly high after graft rejection and gene therapy for SCD. Currently, we are unable to reliably assess genetic risk factors for leukemia development after curative therapies for SCD. Given our current knowledge, we recommend counseling patients about leukemia risk and discussing the importance of an individualized benefit/risk assessment that incorporates leukemia risk in patients undergoing curative therapies for SCD.

## 1. Introduction

Sickle cell disease (SCD) is an autosomal recessive disorder that is one of the most common and severe monogenic diseases worldwide [1]. Affected individuals suffer from acute and chronic disease complications, including pain events, stroke, and organ damage, culminating in early mortality. Several disease-modifying drugs and chronic transfusion therapies are available, but these interventions are not curative. Hematopoietic cell transplantation (HCT) offers a curative option for patients with SCD. The risk of leukemia development increases in individuals with SCD, particularly in specific curative therapy settings. Early data suggest that clonal hematopoiesis may be more prevalent in patients with SCD [2] and that clonal hematopoiesis may expand prior to leukemia development [3]. This review discusses risk factors for clonal hematopoiesis, leukemia development after various curative therapy strategies, and why patients may be at increased risk for leukemia after curative therapy for SCD. 

### 1.1. Pathophysiology of SCD

**Figure 1:**
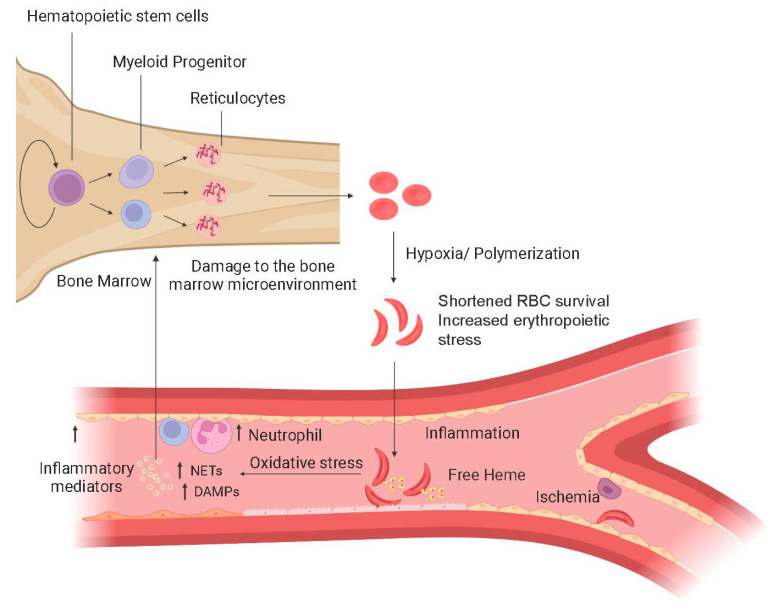
Aspects of SCD pathophysiology that may contribute to clonal hematopoiesis in SCD. Proliferative stress: shortened RBC survival may contribute to high hematopoietic stem cell turnover. Genotoxic stress: free heme, oxidative stress, and other inflammatory mediators damage DNA. Clonal selection: the inflammatory environment may select for clones able to tolerate an unfavorable bone marrow microenvironment. Three processes drive chronic inflammation in SCD: (1) RBC abnormalities; sickle red blood cells (sRBC) damage the endothelium directly through mechanical injury and adhesion. (2) Release of free hemoglobin and heme secondary to sRBC chronic hemolysis; fragile, rigid sRBC have a shortened lifespan, 20 days compared to 120 days. Free hemoglobin and heme result in chronic inflammation, oxidative stress, and vascular damage. The abnormal sRBCs and activated endothelial cells produce a proinflammatory environment; circulating leukocytes and platelets also have an activated phenotype. (3) Ischemia-reperfusion injury secondary to ongoing, intermittent microvascular occlusions promotes chronic inflammation in SCD [8]. Ischemic cells accumulate calcium, exhibit mitochondrial dysfunction and cell swelling, and release major inflammatory-damage-associated molecular patterns (DAMPs) that promote multiple inflammatory pathways, including neutrophil extracellular trap formation and inflammasome assembly [9]. Reperfusion causes further damage due to the production of reactivated oxygen species and calcium overload. Ischemia/reperfusion injury activates invariant natural killer T cells, which trigger interferon gamma (IFN-γ) and IFN-γ-inducible chemokines [10]. Upward arrow indicates levels are higher in SCD than in healthy individuals.

SCD is caused by a point mutation in the sixth position of the β-globin gene; a hydrophilic amino acid, glutamate, is replaced by a hydrophobic valine residue [4]. The result is abnormal hemoglobin (Hb), HbS, which polymerizes under hypoxic conditions to hide the valine residue from the aqueous environment of the red cell. Polymerization exacerbates sickle red blood cell (RBC) rigidity and poor deformability, shortening red cell survival and increasing erythropoietic turnover [5] (Figure 1). Chronic inflammation and stress erythropoiesis negatively impact the SCD bone marrow [6] and may contribute to an increased risk of hematologic malignancies [7].

Chronic inflammation drives many clinical complications experienced by individuals with SCD; it also impacts the bone marrow, causing stress erythropoiesis due to rapid RBC turnover and ineffective erythropoiesis from the apoptosis of erythroid precursors [11]. Polymerization of HbS in erythroid precursors traps heat shock protein 70 (HSP70) in the cytoplasm, preventing it from traveling to the nucleus to protect GATA1 and prevent apoptosis of erythroid precursors [12]. The short lifespan of erythrocytes in SCD creates a sustained need for increased RBC production and, presumably, a need for increased hematopoietic stem and progenitor cells (HSPCs) that imposes significant proliferative stress on the HSPC compartment. 

### 1.2. Morbidity and Mortality in SCD

Individuals with SCD experience high morbidity, beginning in early childhood [13]. Infants under the age of 1 year develop significant splenic dysfunction, causing them to be immunocompromised; in low-resource countries, this is a major cause of mortality in children with SCD under the age of 5 [14]. In high-resource countries, prophylactic penicillin and early medical attention for febrile illness largely prevent sepsis-related death in early childhood. Severe pain events requiring parenteral opioid therapy occur in early childhood and worsen in frequency and duration as individuals enter adulthood. Half of all adults with SCD develop chronic, daily pain, typically poorly responsive to opioids [15]. 

Complications that may result in early mortality include stroke, acute chest syndrome, severe anemia, alloimmunization, pulmonary hypertension, obstructive lung disease, and sickle nephropathy [16,17,18,19,20,21]. In 1994, the life expectancy of individuals with sickle cell anemia (SCA, HbSS, or HbSβ^0^) living in the United States was 45 years for men and 47 years for women. Individuals with HbSC had a life expectancy of 67 years [22]. A follow-up study analyzing data up to 2005 did not show a substantial change in adult survival since the 1994 assessment while capturing improved survival of children with SCD into adulthood, similar to other reports [23,24]. The failure to improve life expectancy in adults with SCD is concerning and demonstrates the need for therapies with curative intent. 

### 1.3. Palliative Therapies in SCD 

Hydroxyurea has been used in SCD for over 30 years, based on its ability to increase fetal hemoglobin (HbF) levels, reduce white blood cells, decrease the adhesion of blood components to the endothelium, and reduce inflammation [25]. Long-term follow-up studies have failed to show an increase in malignancy rates in individuals with SCD exposed to hydroxyurea. In-vitro assays of DNA damage from hydroxyurea use have produced conflicting results [26]. Hydroxyurea is the safest and most efficacious disease-modifying therapy for individuals with SCD, but up to half of adults with SCD do not report significant clinical improvement [25]. In the last five years, three additional drug therapies have been FDA approved for use in SCD. L-Glutamine, which increases the proportion of the reduced form of nicotinamide adenine dinucleotides in sRBCs and may reduce oxidative stress, modestly reduces pain events [27]. Voxelotor reversibly binds to the alpha globin subunit to stabilize HbS in the oxygenated state and prevents polymerization and has been shown to increase hemoglobin and decrease markers of hemolysis [28]. The impact on bone marrow and ability to reduce genotoxic stress with long-term use is unknown [28]. Crizanlizumab is a humanized monoclonal antibody that binds to P-selectin to prevent adhesion of leukocytes and sRBC to the endothelium, reducing vaso-occlusion that leads to ischemia and pain events [29]. Red blood cell transfusions are also employed in acute circumstances, such as patients with stroke, acute chest syndrome, or multi-organ failure, or chronically, such as for primary or secondary stroke prevention. However, complications, including delayed hemolytic transfusion reactions, alloimmunization, and iron overload, may occur [30]. Overall, the effectiveness of these drug therapies to modify SCD varies from individual to individual and they are palliative, not curative.

### 1.4. Curative Therapies for SCD

The only curative option for SCD remains HCT. The most common approach is allogeneic HCT. Traditionally, only children with an HLA-identical sibling underwent HCT [31]. Patients received myeloablative chemotherapy to replace recipient hematopoiesis fully with donor HSCs. Gluckman and colleagues reported this myeloablative approach to result in a five-year overall survival of 95% and an event-free survival of 93% in patients with SCD less than 16 years of age; 14% of all patients developed acute and chronic graft-versus-host disease (GVHD) [32]. 

Unfortunately, due to subclinical and overt organ damage, many adults with SCD cannot tolerate myeloablative conditioning. An alternative approach developed by investigators at the National Institutes of Health (NIH) employs non-myeloablative conditioning, which could be used in patients with severe organ damage [33]. Historically, their goal has been to induce a mixture of donor and recipient hematopoiesis, a state called mixed chimerism. Indeed, 20% donor myeloid chimerism has been reported to be sufficient to reverse the acute complications of SCD [34,35]. Recently, investigators reported 122 patients, primarily adults, who underwent non-myeloablative HLA-matched sibling HCT at one of three centers [36]. At a median follow-up of five years, overall survival was 95% and event-free survival 85%. Two patients developed ≤ Grade 2 acute and no patients chronic GVHD.

While HLA-matched sibling HCT for adults and children is efficacious, only 10–15% of individuals with SCD have an HLA-matched sibling donor [37]. However, 90% of individuals with SCD have a haploidentical (half-matched) parent, child, or sibling who could serve as a donor [38]. The early trials were limited by a high incidence of graft rejection [38,39] and GVHD [40]. However, more recent studies that include post-transplant cyclophosphamide show high efficacy with a low incidence of GVHD [41,42,43,44,45].

Recently, two alternative options have become available as curative options for individuals with SCD: gene therapy and gene editing. With both options, genetically corrected autologous HSPCs are infused into the patient after myeloablative conditioning. Gene therapy employs a viral vector to transport a normal gene into HSPCs. Most experience has been with the bluebird bio-sponsored gene therapy trial. Patients receive autologous HSPCs transduced with a lentiviral vector encoding a modified β-globin gene that produces HbA^T87Q^, which is designed to increase oxygen affinity and reduce sickling. Investigators recently reported the results of 35 patients in the most optimized group [46]. With a median follow-up of 17.3 months, median hemoglobin increased from 8 g/dL at baseline to at least 11 g/dL through 3 years post-gene therapy. Remarkably, in the 25 evaluable patients, all were free of severe vaso-occlusive episodes. 

With gene editing, on the other hand, methods such as clustered regularly interspaced palindromic repeats-associated protein-9 nuclease (CRISPR-Cas9) are used to cut the abnormal gene and then replace it with a normal gene. Gene editing studies are just getting underway, with impressive early results [47]. Notably, gene therapy and gene editing require myeloablative chemotherapy, limiting these options to a minority of adults with SCD. Further, there are no guidelines to inform patients or providers about which curative therapy option each patient should pursue. 

## 2. Clonal Hematopoiesis

### 2.1. Definition, Prevalence, and Clinical Consequences of CH

The acquisition of somatic mutations during cell division is inevitable. On average, two to three new mutations are generated after each cell division due to DNA replication error [48]. The vast majority of these mutations are biologically inconsequential and contribute to somatic mosaicism uniformly present in all tissues. Random mutations rarely affect genes whose products play a key role in stem cell self-renewal and differentiation programs. Even subtle changes in this strictly controlled process may increase HSC fitness and clonal outgrowth of a genetically distinct population of cells. In hematopoietic tissue, such dominance of mutation-carrying cells over unmutated counterparts is known as clonal hematopoiesis (CH). It is also not surprising that the lion’s share of genes frequently mutated in CH is also associated with hematologic malignancies. The HSC fate seems to be controlled mainly at the epigenetic level, either through DNA methylation or chromatin modification. Therefore, it is not a coincidence that genes encoding *DNMT3A* and *TET2* enzymes involved in DNA methylation and *ASXL1*, an important regulator for the chromatin state, account for >90% of CH [49,50]. 

The number of somatic mutations increases with age, and CH appears to be a common age-related condition. While CH is exceedingly rare in people under the age of 40 years, its prevalence increases with each decade of life, reaching nearly 40% in individuals 60 years and older [51]. However, the prevalence of CH greatly depends on the characteristics of the molecular assays applied to detect somatic mutations. On one end, targeted sequencing approaches are limited to a handful of leukemia-associated genes, and suboptimal depth, even though highly specific for CH, may be insufficient to capture a significant proportion of individuals with CH. On the opposite end of the spectrum, ultra-deep or extensive breadth next-generation sequencing (NGS) methods often identify a large proportion of variants with unknown biological significance. The majority of these variants represent ubiquitous somatic mosaicism; their presence is often clinically and biologically irrelevant and does not indicate a clonal process [52,53]. 

Since mutations in leukemia-associated genes are predominant in CH, it is not surprising that some clones may eventually give rise to clinically apparent hematologic malignancy. Fortunately, less than 1% of CH-affected individuals will suffer this dreadful consequence. CH is a vastly heterogeneous entity, and its malignant potential is determined by the type and the number of mutated genes and the size of the clone. For instance, in native CH, the presence of mutations in spliceosome genes, *IDH1/2*, *TP53*, or multiple mutations, and high-variant allele frequency (VAF) are associated with an increased risk of subsequent acute myeloid leukemia (AML) or myelodysplastic syndrome (MDS). Interestingly, the risk of leukemic transformation does not appear to be significantly increased when only a single *DNMT3A* or *TET2* mutation is present [54,55]. These findings were recently confirmed in allogeneic HCT, where recipients received the graft from a donor with CH. Donor-cell leukemia developed only in recipients whose donors had multiple mutations, mutations in spliceosome machinery, *TP53*, or germline *DDX41* mutation. Interestingly, none of the recipients whose donors had sole *DNMT3A* or *TET2* mutations developed DCL [51].

Unlike in native CH, where driver mutations impose a predominantly cell-autonomous effect, specific mutations may increase HSC fitness explicitly in the presence of an extracellular stressor. Patients treated with chemo- or radiotherapy are at risk for MDS or leukemia. Minute clones carrying *TP53* or *PPM1D* mutations, frequently undetectable by standard NGS techniques, are present long before treatment with genotoxic agents [56]. These mutations are often functionally silent under homeostatic conditions but can evade apoptosis triggered by genotoxic stress and acquire additional DNA defects. For example, the loss of a wild-type *TP53* allele in a patient with heterozygous *TP53* mutations may result in complete loss of function and subsequent accumulation of additional genetic defects and leukemic transformation [57,58]. Thus, the malignant potential of CH seems to be independent of clonal size and instead determined by the type of mutations and biological context. 

In addition to its malignant potential, CH has also been associated with adverse outcomes, particularly an increased risk of cardiovascular events. This sequela seems to be driven predominantly by overexpression of proinflammatory cytokines and chemokines in *DNMT3A* or *TET2*-mutated monocytes/macrophages. Moreover, the impact of CH on ischemic cardiovascular events is more evident in patients with significantly expanded CH clones, specifically with VAF > 10% (>20% clonal HSC). Thus, distinct CH genotypes and clonal burdens may determine diverse clinical consequences. 

### 2.2. Risk Factors for Clonal Hematopoiesis

Age is by far the most significant risk factor for CH. Other risk factors include ionizing radiation, cytotoxic chemotherapy, particularly platinum agents, smoking, obesity, and male sex [59]. The impact of race on CH is less clear. Some studies demonstrated that CH was less common in persons of Hispanic ancestry, and no significant differences were found between Caucasians and African Americans [50,60]. Even though chronic inflammation and infection have been associated with CH, it is unclear whether they increase somatic mutagenesis in HSCs or impose extracellular constraints on healthy HSCs, allowing for selective expansion of preexisting mutated clones [61,62]. Alternatively, chronic inflammation may increase the proliferation rate of HSC, resulting in accelerated CH [63]. 

Chronic hyperproliferative anemia resulting in rapid hematopoietic cell turnover, systemic inflammation, and frequent infections are the hallmark of SCD. Increased HSC proliferation fueled by hemolytic anemia and inflammation may significantly accelerate somatic mutagenesis and clonal expansion of mutated clones. Thus, it is plausible that CH is more common and occurs early in SCD due to accelerated biological aging of the hematopoietic system. 

### 2.3. Clonal Hematopoiesis and Sickle Cell Disease

Due to an increased risk of leukemia in individuals with SCD (see below), reports are coming forth and studies are ongoing to evaluate the prevalence of CH in SCD. Because of an increased incidence of leukemia in patients with CH with a VAF of at least 10% [49,50], Liggett and colleagues recently evaluated almost 75,000 whole genome sequencing analyses from 3090 individuals with SCD and 71,100 individuals without SCD [64]. Clonal hematopoiesis was reliably evaluable in clones with a VAF of >10% and in about half of the clones with a VAF of 5–10%. The prevalence of CH in this study was not significantly different between subjects with SCD and the control group.

To assess CH at a deeper level, Pincez et al. assessed whole exome sequencing data from 1459 patients with SCD and 6848 African-American controls; 517 had sickle cell trait [2]. Variant allele frequency ranged from 2.5 to 26%, with a median of 7%. Even though the cohort with SCD was younger (mean age 23.6 years versus 50.1 years), CH was more prevalent in individuals with than without SCD. When accounting for variables, including age and sex, SCD was independently associated with an increased rate of CH (odds ratio 13.5, 95% confidence interval 3.1–41.9). Interestingly, *TP53*, which is infrequently affected in the healthy population, was the second most commonly mutated gene (after *DNMT3A*) and accounted for 13% of all CH mutations in SCD.

### 2.4. Clonal Hematopoiesis and Curative Therapies for Sickle Cell Disease

Clonal hematopoiesis mutations have also been reported in individuals with SCD who develop leukemia after curative therapies. Of 15 patients with SCD who developed leukemia, with 2 having undergone HCT, somatic mutation analysis was performed in 6 patients [65]. Four patients had *TP53* mutations at the time of leukemia diagnosis. Ghannam and colleagues recently reported that 3 of 76 patients with SCD developed leukemia after rejecting their grafts [3]. Two patients had *TP53* mutations present at leukemia diagnosis at VAF ranging from 2.9% to 24.3%. The authors queried whether the somatic mutations were caused by the chemotherapy and total-body irradiation conditioning or whether the somatic mutations were already present at baseline due to a lifetime of erythropoietic stress and inflammation. The exact pathogenic *TP53* mutations were present at baseline but at much lower levels, with VAF ranging from 0.03 to 0.35%.

Somatic mutation abnormalities were also reported in two patients who developed leukemia after gene therapy for SCD [66,67]. Interestingly, unlike in the allogeneic setting, *TP53* mutations were not found. Instead, both patients had *RUNX1* and *PTPN11* mutations and monosomy 7 chromosomal abnormalities. While NGS was performed in both patients at baseline, ultradeep sequencing has not been reported to assess whether the same somatic mutations were present prior to gene therapy conditioning.

## 3. Leukemia and Sickle Cell Disease

### 3.1. Risk of Leukemia in Sickle Cell Disease

Two large population studies have reported an increased risk of leukemia in patients with SCD [68,69]. The first included 7512 individuals with SCD who were admitted or died between 1999 and 2011 compared to 118,821 African-American controls [69]. They found a 2.1-times higher risk for all cancers combined and a 3.3-times higher risk for lymphoid leukemia. However, there was an 11.1-times higher risk for AML. The second study analyzed 6423 patients with SCD, identified between 1991 and 2014, who were followed for 141,752 person-years compared to the general population [68]. They did not detect an increased incidence of solid tumors. However, they found a 2.3-fold increased risk for leukemia and a 3.6-fold increased risk for AML. 

Additional analyses were performed to assess why individuals with SCD have an increased risk for leukemia [68]. While there was no difference in sex between patients with SCD who did versus did not develop leukemia, patients aged 15 to 39 years were more likely to develop leukemia than those under 15. Further, those with more severe disease, defined as patients with at least three hospitalizations or emergency room visits per year, were more likely to have leukemia than those with less severe disease. Finally, the authors compared the prevalence of leukemia from 1988 to 1999 to the prevalence of leukemia between 2000 and 2014. Importantly, the prevalence of leukemia did not increase in the latter treatment cohort, supporting, as above, that hydroxyurea does not appear to increase leukemia risk in individuals with SCD. 

Further, while both studies showed an increased risk of leukemia in individuals with SCD, the number of patients in each study who developed leukemia was low (Table 1). For example, as stated above, Brunson and colleagues compared 6423 patients with SCD to the general population with 23 years of data [68]. While patients with SCD had a 3.6-fold increased risk for AML, 6 patients developed AML compared to 1.67 expected when adjusted for age, sex, race, and ethnicity. Therefore, while the relative risk for leukemia development is high in individuals with SCD, the absolute risk is low.

### 3.2. Risk of Leukemia after Curative Therapies for SCD

The risk of leukemia is higher than expected based on the modestly increased risk in untransplanted patients with SCD after specific curative therapies for SCD (Table 1). As previously mentioned, Li and colleagues reported two patients who developed leukemia after allogeneic HCT for SCD [65]. Others have reported leukemia after graft rejection for SCD [39,74]. The NIH group reported 3 of 76 patients (4%) who developed leukemia after non-myeloablative allogeneic HCT for SCD; all 3 patients had rejected their grafts [34]. Further, a multi-center study, including the University of Illinois Chicago and King Abdulaziz Medical City in Riyadh, Saudi Arabia, reported 1 of 64 patients (1.6%) who developed leukemia after HLA-matched sibling HCT using the NIH regimen with a median follow-up of up to four years [36]; the patient rejected the graft. As the aforementioned leukemias occurred after rejecting the graft, the leukemia appears to be recipient-derived. Moreover, 2 of 47 patients (4.3%) developed leukemia after gene therapy for SCD [66,67]. Notably, an extensive evaluation revealed that leukemia was not directly related to the vector in both cases. Lastly, the group from Belgium reported 1 of 50 patients (2%) who developed leukemia after myeloablative HLA-matched HCT for SCD [71]. The leukemia was found to be of donor origin. The association between CH in donors and donor-cell leukemia has been well-established [75].

These high incidence rates of leukemia compare unfavorably to a much lower incidence of leukemia reported in large multi-center trials of allogeneic HCT for SCD (Table 1). A study based on registry data reported 910 patients who underwent allogeneic HCT at 1 of 90 United States centers between 2008 and 2017 [73]. Five patients (0.55%) developed leukemia. In addition, a French group recently reported 234 patients with a median age of 7.9 years who underwent HLA-matched HCT at various sites between 1988 and 2012 [72]. Seventy-nine patients had mixed long-term chimerism. None of the patients developed leukemia.

## 4. Why Is the Risk of Leukemia Increased in Patients with SCD after Curative Therapies?

**Figure 2:**
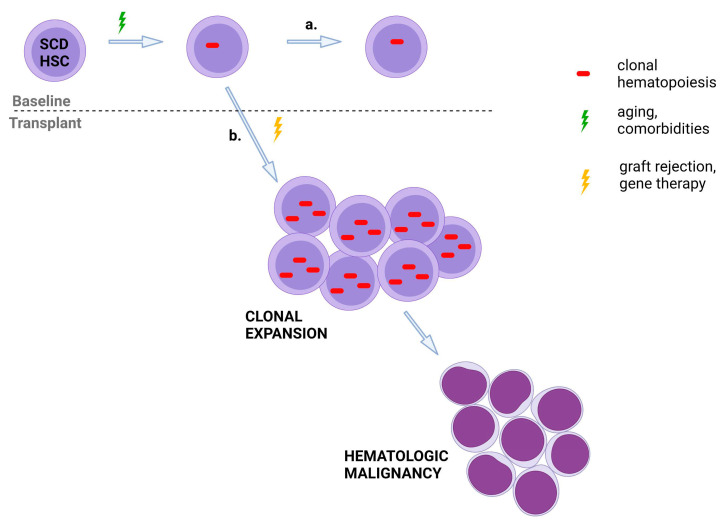
Theory for why curative therapy requiring regenerative hematopoiesis has a higher incidence of leukemia. a. Individuals with SCD may develop low-level CH that does not expand and progress to leukemia over time. b. After graft rejection or gene therapy, the pressure of changing from homeostatic to regenerative hematopoiesis by autologous cells drives clonal expansion and leukemogenic transformation of preexisting premalignant clones, eventually resulting in leukemia. SCD: sickle cell disease; CH: clonal hematopoiesis; HSC: hematopoietic stem cell. Created with BioRender.com (accessed on 14 April 2022).

Why is the incidence of leukemia higher than expected in patients who have rejected their grafts and after gene therapy for SCD compared to individuals who do not receive curative therapy for SCD? Interestingly, while the duration of follow-up is limited, leukemia has only been diagnosed in Group A instead of Group C patients [46,66,67]. The conditions prior to drug product infusion with Group C patients were optimized, including a requirement for exchange transfusions and peripheral blood stem cells instead of bone marrow as the HSC source in Group C patients [46,67]. Group C patients maintained long-term repopulating HSPCs, which supported RBC production with an increase in median hemoglobin, from 8.5 to at least 11.0 g/dL, through 36 months post-gene therapy [46]. Conversely, similar to graft rejection in the allogeneic setting, Group A gene therapy patients depended on a low number of HSPCs to repopulate the bone marrow [67] (Figure 2). Therefore, autologous reconstitution in both cases is associated with substantial proliferative stress. After graft rejection and gene therapy, the stress of changing from homeostatic to regenerative hematopoiesis by autologous and transduced HSC may drive clonal expansion and leukemogenic transformation of preexisting premalignant clones, ultimately leading to AML or MDS [70]. 

Moreover, a population of unmanipulated autologous HSCs may survive preparative conditioning with genotoxic chemotherapy, irradiation, or both. This extracellular stress may further impose a selection pressure favoring premalignant clones with already disrupted DNA-damage repair pathways [59]. Like MDS or AML arising after autologous HCT, preexisting clones carrying the *TP53* or *PPM1D* mutation may give rise to MDS or AML in patients after gene therapy or in recipients of allogeneic HCT who rejected their grafts. However, the prevalence of primed premalignant clones in SCD patients is unclear. Since the two large population studies above reported an increased relative risk of leukemia in patients with SCD and whole exome sequencing analysis showed an increased prevalence of somatic mutations in individuals with SCD, there is evidence suggesting that premalignant CH may be more common in this population. Finally, perhaps the most convincing evidence comes from the NIH case series of recipients of allogeneic HCT discussed above, who developed AML after graft failure. In two cases, *TP53* mutations were present before HCT and evolved within 3 years post-transplant [3]. Of note, in both cases, *TP53* mutant clones accounted for <1% of the total hematopoietic output, supporting the notion that the nature of mutations and biological context, rather than clone size, determine the oncologic potential of CH. Assessment of low-level CH in individuals with SCD is critically needed. 

## 5. How to Counsel Patients about Baseline Risk of Leukemia after Curative Therapies for Sickle Cell Disease—Our Opinion

Clonal hematopoiesis is now a well-recognized leukemia predisposition syndrome. In the general population, CH has been associated with a 5 to 10-fold increase in myeloid malignancies [50]. Preexisting CH, particularly clones with mutations in DNA-damage response genes, such as *TP53* or *PPM1D*, may evolve into myeloid malignancies. In the allogeneic HCT setting, autologous premalignant clones may evade genotoxic conditioning and acquire additional leukemogenic mutations, leading to malignant transformation and clinically apparent myeloid malignancy. Therefore, patients with autologous hematopoietic recovery after allograft rejection may be at particular risk for this often-lethal complication [3]. Similarly, pre-existing CH or mutations acquired during ex-vivo manipulation and gene editing of HSPCs may set the stage for subsequent myeloid malignancies that can be further potentiated by genotoxic conditioning. Thus, the presence of CH may guide the therapeutic decisions in patients with SCD. Perhaps individuals with SCD and high-risk CH (*TP53*, *PPM1D* mutations) should be offered curative therapies intended to fully eradicate leukemic-prone hematopoiesis. The contemporary allogeneic HCT approaches utilizing optimized conditioning strategies frequently lead to full donor chimerism. Alternatively, patients without detectable CH or hereditary cancer predisposition syndromes would be most suitable for novel gene therapy or gene editing therapies. These approaches are likely to be refined as we learn more about the malignant potential of various somatic mutations and better understand the effect of cell processing during gene therapy or gene editing on leukemogenesis. 

## 6. Conclusions

While whole genome sequencing did not show an increased prevalence in CH [64], deeper whole exome sequencing revealed a higher prevalence of CH in patients with SCD compared to healthy controls [2]. Though the relative risk of leukemia is higher in individuals with SCD, the absolute risk is low. Conversely, the incidence of leukemia is higher than expected after graft rejection and gene therapy for SCD; the reasons are likely multi-factorial. No reliable baseline screening studies are currently available to assess the risk of leukemia development after curative therapies for SCD, but this is an active area of research, and international registries and biospecimen banking are needed. In addition, a benefit/risk assessment is critical to balance the risk of leukemia to the risk of early death from SCD itself.

## Figures and Tables

**Table 1:** Percentage of Patients Reported with Myelodysplastic Syndrome or Leukemia in Individuals with Sickle Cell Disease with and without Curative Therapy.

Reference	Sample Size	HCT Type	Conditioning Agents	Number (%) with MDS or Leukemia	Time to MDS/Leukemia Diagnosis
Ghannam et al. [3]	76	HLA-matched sibling Haploidentical	Alemtuzumab 300–400 cGy TBI PT-Cy 0–100 mg/kg	3 (3.9%)	2–5 years
Jones and DeBaun [70]	47	Gene therapy	Busulfan	2 (4.3%)	3–5.5 years
Vermylen et al. [71]	50	HLA-matched family member	Busulfan Cy ±r-ATG ±TLI	1 (2%)	35 months
Bernaudin et al. [72]	234	HLA-matched sibling	Busulfan Cy r-ATG	0 (0%)	N/A
Eapen et al. [73]	910	Mostly HLA-matched sibling	Mostly Busulfan Cy r-ATG	5 (0.55%)	9 to 44 months
Seminog et al. [69]	7512	N/A	N/A	<35 (<0.47%)	Observed over 12 years
Brunson et al. [68]	6423	N/A	N/A	12 (0.19%)	Observed over 23 years

Abbreviations: HCT: hematopoietic cell transplant, MDS: myelodysplastic syndrome, HLA: human leukocyte antigen, cGy: centigray, TBI: total body irradiation, PT-Cy: post-transplant cyclophosphamide, Cy: cyclophosphamide, r-ATG: rabbit-anti-thymocyte globulin, TLI: total lymphoid irradiation; N/A: not applicable.

## Data Availability

Not applicable.
